# Sonoporation based on repeated vaporization of gold nanodroplets

**DOI:** 10.1002/mp.15544

**Published:** 2022-03-03

**Authors:** Wei‐Wen Liu, Hung‐Chih Ko, Pai‐Chi Li

**Affiliations:** ^1^ Graduate Institute of Biomedical Electronics and Bioinformatics National Taiwan University Taipei Taiwan; ^2^ Department of Electrical Engineering National Taiwan University Taipei Taiwan

**Keywords:** acoustic droplet vaporization, gold nanodroplets, inertial cavitation, optical droplet vaporization, sonoporation

## Abstract

**Background:**

Gold nanodroplets (AuNDs) have been proposed as agents for photothermal therapy and photoacoustic imaging. Previously, we demonstrated that the sonoporation can be more effectively achieved with synchronized optical and acoustic droplet vaporization. By applying a laser pulse at the rarefactional phase of the ultrasound (US) pulse, the vaporization threshold can be reached at a considerably lower laser average power. However, a large loading quantity of the AuNDs may increase the risk of air embolism. The destruction of phase‐shifted AuNDs at the inertial cavitation stage leads to a reduced drug delivery performance. And it also causes instability of echogenicity during therapeutic monitoring.

**Purpose:**

In this study, we propose to further improve the sonoporation effectiveness with repeated vaporization. In other words, the AuNDs repeatedly undergo vaporization and recondensation so that sonoporation effects are accumulated over time at lower energy requirements. Previously, repeated vaporization has been demonstrated as an imaging contrast agent. In this study, we aim to adopt this repeated vaporization scheme for sonoporation.

**Methods:**

Perfluoropentane NDs with a shell made of human serum albumin were used as the US contrast agents. Laser pulses at 808 nm and US pulses of 1 MHz were delivered for triggering vaporization and inertial cavitation of NDs. We detected the vaporization and cavitation effects under different activation firings, US peak negative pressures (PNPs), and laser fluences using 5‐ and 10‐MHz focused US receivers. Numbers of calcein‐AM and propidium iodide signals uptake by BNL hepatocarcinoma cancer cells were used to evaluate the sonoporation and cell death rate of the cells.

**Results:**

We demonstrate that sonoporation can be realized based on repeatable vaporization instead of the commonly adopted inertial cavitation effects. In addition, it is found that the laser fluence and the acoustic pressure can be reduced. As an example, we demonstrate that the acoustic and optical energy for achieving a similar level of sonoporation rate can be as low as 0.44 MPa for the US PNP and 4.01 mJ/cm^2^ for the laser fluence, which are lower than those with our previous approach (0.53 MPa and 4.95 mJ/cm^2^, respectively).

**Conclusion:**

We demonstrated the feasibility of vaporization‐based sonoporation at a lower optical and acoustic energy. It is an advantageous method that can enhance drug delivery efficiency, therapeutic safety and potentially deliver an upgraded gene therapy strategy for improved theragnosis.

## 1 INTRODUCTION

Phase‐changeable nanodroplets (NDs) are getting more noticed on tumor therapy due to their smaller size and longer circulating life. How to reduce the unwanted harm of healthy cells caused by the high driving energy for inducing vaporization, inertial cavitation, and further sonoporation is still a goal to pursue. Vaporization of droplets can be achieved through optical and/or acoustic activation. Delivery of ultrasound (US) waves has been reported to be effective to induce acoustic droplet vaporization (ADV), with which the peak negative pressure (PNP) at the rarefaction phase is sufficient to lower the ambient pressure surrounding the droplets or to induce a temperature elevation surpassing the threshold for the phase transition.^1^, ^2^, ^3^ On the other hand, either the continuous wave laser or the pulsed wave laser can induce optical droplet vaporization (ODV). ODV threshold has been reported to be positively correlated with the size of droplets, where smaller droplets require lower laser fluence for phase transition.^4^ Except droplet size, many parameters have also been investigated to relate to the vaporization threshold including temperature, driving US frequency and intensity, and shell properties.^5^, ^6^, ^7^ In the literature, vaporization of nanosized perfluoropentane (PFP)‐based droplets requires vaporization temperature above 73.2°C,^8^ and driving acoustic pressure above 1.05 MPa,^9^, ^10^, ^11^ which may cause unwanted tissue damage in some cases.

The simultaneous application of US and continuous or pulsed laser has been reported to facilitate a reduced vaporization or cavitation threshold and thus help expand the biomedical applications to imaging, therapy, and drug delivery.^12^, ^13^, ^14^, ^15^ By properly synchronizing the pulsed laser and the US, it has been demonstrated that image contrast, sonoporation, and therapeutic performance can be greatly improved.^14^, ^15^ The sono‐photoacoustic (PA) method developed by simultaneously transmitting US and laser not only significantly enhances the PA imaging contrast, but also successfully reduced the vaporization threshold.^12^, ^14^, ^16^, ^17^, ^18^, ^19^ Repeatable vaporizations have also been reported for imaging that can be attained either through ODV or through a combination of ADV and ODV.^8^, ^20^, ^21^, ^22^ For example, repeatability of ODV of droplets ranging from 0.2–1.0 μm has been reported to be size‐dependent, where larger droplets produce stronger PA signals, but compared to the PA signal intensity generated through thermal expansion, even the small droplets are capable of producing three times larger PA signal intensity.^20^

Our previous study proposed that the laser and US can be synchronized by applying the laser pulse at the rarefactional phase of the US pulse.^15^ The synchronization enables effective sonoporation using gold nanodroplets (AuNDs) in a more controlled manner. We demonstrated that the concurrently triggered optical and acoustic pulses were essential to induce vaporization and the subsequent inertial cavitation of AuNDs. Our results suggested that when delivering specific optical energy to AuNDs, the vaporization of AuNDs and the subsequent inertial cavitation can be repeatedly induced during the activation process. Moreover, under the same optical parameters, the vaporization of AuNDs can be enhanced by using larger PNP without inducing inertial cavitation, suggesting that the delivery of acoustic energy to AuNDs is more capable of controlling the induction of vaporization instead of inertial cavitation. According to the literature, based on the images captured by a high‐speed camera, the size of vaporized ND is increased by dozens of times larger than the NDs in the liquid phase.^23^ The vaporized ND can then condense back to the liquid phase within microseconds following excitation.^23^, ^24^ Due to the fact of repeatable volumetric change occurred during the phase transition stage of NDs,^25^ repeatable vaporization‐recondensation processes may become a new mechanism potentially for more effective sonoporation. We thus hypothesize that through properly adjusting the laser/US activation firings and the associated energy, repeated vaporization of AuNDs can be achieved. In other words, the same AuNDs can be repeatedly used during sonoporation without destruction due to inertial cavitation. In addition, the driving optical and acoustic energy levels can be further reduced for sonoporation.

For in vivo applications, a less loading quantity of AuNDs can potentially reduce the possibility of induction of air embolism during sonoporation‐based therapy. Decreased energy threshold of sonoporation without induction of inertial cavitation during the sonoporation process not only improves the drug delivery efficiency but the therapeutic safety as well. Moreover, repeated vaporization of AuNDs enhances the PA signal intensity as the AuNDs are often used as the PA contrast agent.^8^, ^20^, ^21^, ^22^ A successful cavitation‐based gene therapy depends on producing the gene‐transfected cells. However, cavitation has still been generally determined as an effect hard to be controlled and predicted in some cases,^26^ and cell damage typically induced by bioeffects or the mechanical actions of the cavitation bubbles.^27^, ^28^, ^29^, ^30^, ^31^, ^32^, ^33^ We thus aim to explore the possibility of inducing sonoporation based on repeated vaporization to reduce the required driving energy for an improved therapeutic strategy in an efficient and safe manner, and also may improve the performance of image‐guided theragnosis.

## 2 MATERIALS AND METHODS

### 2.1 AuND fabrication and characterization

AuNDs were fabricated as reported in our previous works.^13^, ^15^ 48 × 12 nm^2^ gold nanorods (AuNRs) with a longitudinal absorption peak at 818 nm were utilized as the optical absorbers to generate the photothermal effect. The PFC core component of AuNDs was PFP (C_5_F_12_) with a boiling temperature of 29°C at ambient pressure, and the shell of AuNDs was formed by 20% human serum albumin (HSA; Octapharma AG, Lachen, Switzerland) and avidin (ThermoFisher Scientific, Waltham, MA, USA). To synthesize 1 ml of fluorescein isothiocyanate (FITC)‐CD54‐conjugated AuNDs, 200 μl of 20% HSA, 100 μl of 39.5 nM AuNRs, 80 μl of 10 mg/ml avidin, and 75 μl of PFP were mixed in 545 μl of phosphate‐buffered saline (PBS; purchased from Gibco, Thermo Fisher Scientific). The mixture was then sonicated by using a digital sonifier (Branson, Danbury, CT, USA) with a cup‐horn sonotrode (Branson). After four 5‐min sonication‐rest cycles, the AuNDs emulsions were produced. The precipitated AuNDs were then resuspended in 1 ml of PBS followed by three cycles of centrifugation at 1700 rpm for 3 min at 4°C to isolate nano‐scaled droplets. The number and size distribution of droplets were further analyzed by Coulter MultiSizer III (Beckman‐Coulter, Hamburg, Germany) and Zetasizer (Nano Z, Worcestershire, UK), respectively.

For the conjugation of CD54 antibody to AuNDs, 20 μl of FITC‐conjugated biotinylated anti‐CD54 antibody (BioLegend, San Diego, CA, USA) was added into 1 ml size isolated AuNDs, and slightly shaking with 200 rpm at 4°C for 1 h. After the incubation, the mixture was then centrifuged at 2000 rpm for 3 min at 4°C to remove the free antibody left in the supernatant. To confirm the ability of conjugation, AuNDs without antibody conjugation and FITC‐CD54‐conjugated AuNDs were analyzed by flow cytometry. Before doing sonoporation experiments, cells were incubated with FITC‐CD54‐conjugated AuNDs for 30 min at 37°C, 5% CO_2_ incubator. After the incubation, cells were investigated under a fluorescence microscope (IVM‐2A; SAGE Vision, New Taipei City, Taiwan) to ensure the ability to recognize and attach to the cells.

### 2.2 Experimental setup

**FIGURE 1:**
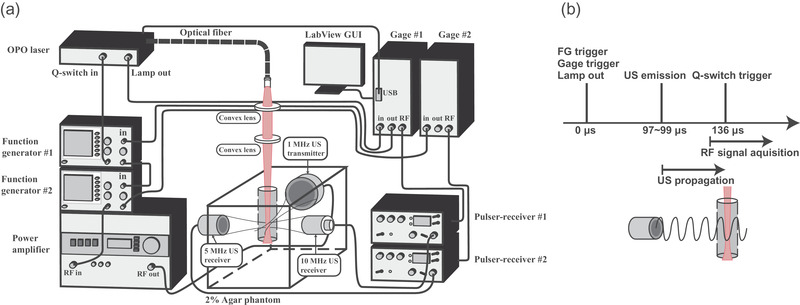
Experimental setup. (a) System setup and (b) time sequence for synchronizing laser and ultrasound pulses. Optical parametric oscillator (OPO) laser indicates pulsed optical parametric oscillator laser. RF signal indicates radio frequency signal. FG indicates the function generator

The experimental setup is schematically shown in Figure 1a, and the time sequence for synchronizing the US and laser activation was illustrated in Figure 1b. They were generally the same as the setups used in our previous study.^15^ But the two receiving US transducers were replaced by the transducers with geometric focusing. The 10‐MHz focused US transducer (V327‐SU; Panametrics‐NDT, Waltham, MA, USA, focused at 30.5 mm) was for receiving cavitation signals and the 5‐MHz focused US transducer (V326‐SU; Panametrics‐NDT, Waltham, MA, USA, focused at 31.7 mm) was used to receive vaporization signals.

We used a wavelength‐tunable optical parametric oscillator (OPO) laser (Opolette 532; OPOTEK, Carlsbad, CA, USA) to produce 808‐nm laser beams with a 10‐ns pulse duration. The laser fluence was measured at the confocal site using a power meter (Nova II; Ophir, Jerusalem, Israel). The diverged laser beam was passed through two plano‐convex lenses to generate a collimated light and a focused laser beam with a Gaussian profile (–6 dB beamwidth = 0.7 mm). The three US transducers and laser beam were co‐focused at the center of a columnar hole formed in a 2% agar phantom.

A 20‐Hz transistor‐transistor logic signal sent from the flash lamp of the laser was used as the system clock. The flash lamp signal was used to trigger the ADC board (CompuScope 14200; Gage, Lockport, NY, USA) controlled by LabVIEW to send a trigger out for triggering two function generators (AFG532; Tektronix, Beaverton, OR, USA and 33522A; Agilent Scientific, Santa Clara, CA, USA). The first function generator was used to produce a 1 MHz, 10‐cycle sine wave delayed from 97 to 99 μs for investigating the signals corresponding to the 8th–10th cycle of US waves. The signal was amplified by a power amplifier (250A250A; Amplifier Research, Souderton, PA, USA) for driving the 1‐MHz focused US transducer (V302‐SU; Panametrics‐NDT, Waltham, MA, USA, focused at 50.8 mm, f# = 1.96). The second function generator was used to send out a trigger delayed by 136 μs to trigger the laser Q‐switch. Signals received from two receiving US transducers were recorded by the ADC at a 100‐MHz sampling rate. All signal and image processing were analyzed in MATLAB (R2019b; The MathWorks, Natick, MA, USA).

### 2.3 Differential vaporization and cavitation doses, and acoustic pressure measurements

**FIGURE 2:**
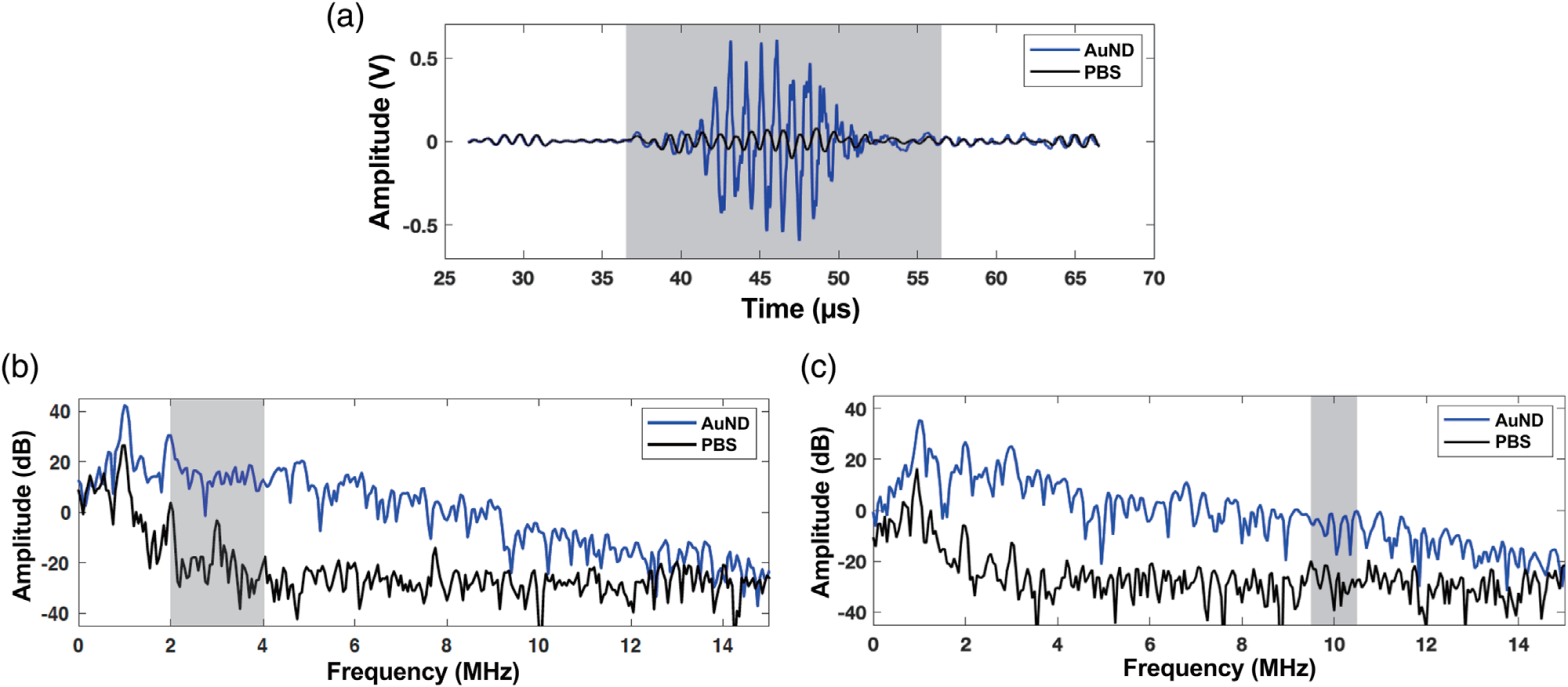
Vaporization and inertial cavitation dose. (a) Representative received signals (b) Spectra of the received vaporization signals. (c) Spectra of the received inertial cavitation signals. Gray boxes indicate the selected regions for data analysis. The baseline signals were received from phosphate‐buffered saline (PBS) only

According to previous studies,^15^, ^34^ we calculated the amplitude of the second to fourth harmonic root‐mean‐square (RMS) values in the frequency domain of the received US signals as the vaporization signals, and the RMS values of the spectrum between 9.5 and 10.5 MHz were calculated as the inertial cavitation signals. Representative received RF signals and the corresponding spectra are shown in Figure 2. After baseline subtraction, the resulting time‐amplitude curve over the entire recording time period was defined as differential inertial cavitation dose (dICD) and differential vaporization dose (dVAP) to represent the occurrence of inertial cavitation and vaporization events, respectively. The PNPs were calculated from the acoustic field generated by a 1‐MHz US transducer, and it was mapped by using a needle‐type hydrophone (MHA9‐150; FORCE Technology, Denmark).

### 2.4 Sonoporation and cell death rate

**FIGURE 3:**
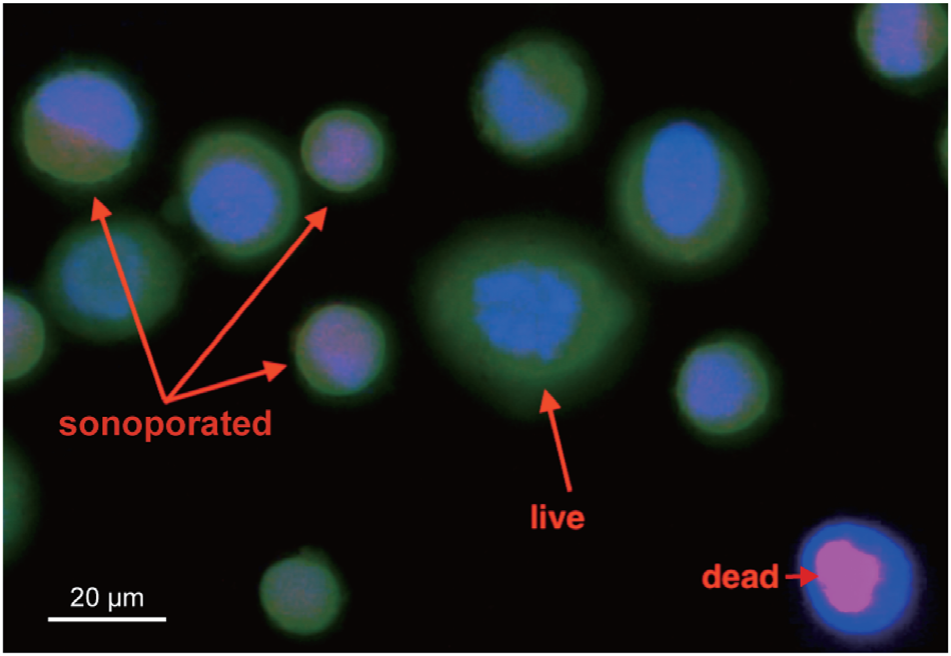
Sonoporation and cell death of the cells. Red fluorescence denotes the propidium iodide (PI) dye. Green fluorescence denotes the calcein‐AM viability dye. Blue fluorescence denotes the cell nuclei. Red arrows indicate examples of sonoporated, live, or dead cells. Scale bar: 20 μm

To better compare the sonoporation data to our previous study,^15^ we used the same type of cell, BNL cell, which is a mouse hepatocarcinoma cancer cell line, as the in vitro cell model. BNL cells were cultured with Dulbecco's Modified Eagle Medium containing 10% fetal bovine serum and 0.1% penicillin/streptomycin (all purchased from Gibco, ThermoFisher Scientific, Waltham, MA, USA) at 37°C, 5% CO_2_ incubator. A membrane impermeant fluorescence dye, propidium iodide (PI) (eBioscience, Thermo Fisher Scientific), was used to monitor the sonoporated cells. Before sonoporation experiments, PI was added into cell suspensions with a 1:50 dilution. Then, cells/PI mixtures were mixed well with AuNDs to make the final concentration of cells and AuNDs to 2 × 10^6^ cells/ml and 2 × 10^8^ droplets/ml, respectively. After sonoporation, cells collected from the hole of the phantom were further counterstained with calcein‐AM viability dye with a 1:2500 dilution (Molecular Probes, Invitrogen, Carlsbad, CA, USA) and a cell nuclear indicator, Hoechst 33342 (NucBlue; Molecular Probes, Invitrogen) with a 1:50 dilution, for 10 min on ice. The sonoporated and dead cells were all calculated under an inverted fluorescence microscope (IVM‐2A; SAGE Vision, New Taipei City, Taiwan). We defined a successful sonoporation as a cell displaying both positive PI and calcein‐AM fluorescence, and if a cell displayed positive PI fluorescence but lacked calcein‐AM fluorescence, the cells would be determined to be subjected to cell death (Figure 3).

## 3 RESULTS

### 3.1 Characterization of AuNDs

**FIGURE 4:**
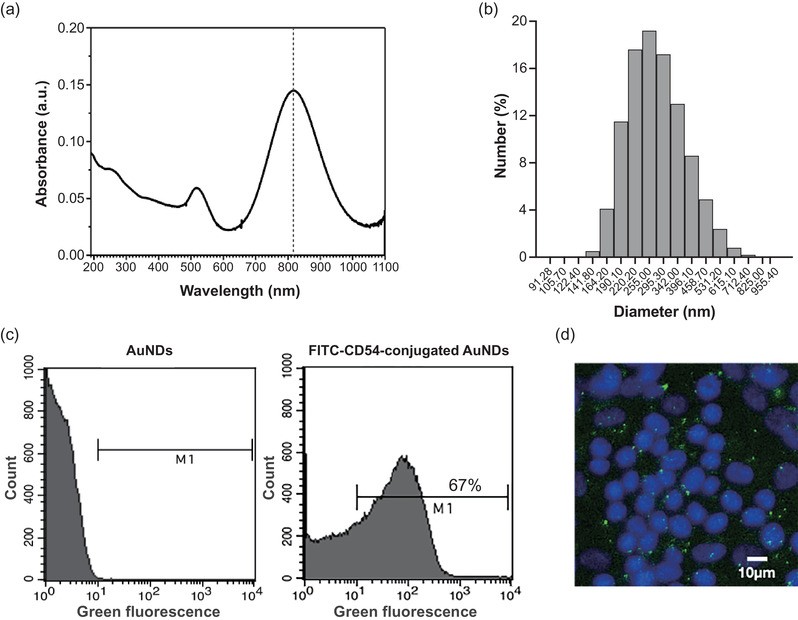
**Characterization of gold nanodroplets (AuNDs)**. (a) The optical absorbance of gold nanorods. The dashed line indicated the peak absorbance of gold nanorods was 818 nm. (b) Size distribution of AuNDs. (c) Flow cytometry results. AuNDs with FITC fluorescence were gated in the M1 region. (d) Fluorescence image of cells attached with FITC‐CD54‐conjugated AuNDs. Green fluorescent dots indicate the FITC‐CD54‐conjugated AuNDs. Blue fluorescence indicates the cell nuclei

By using the emulsification method, we generated NDs encapsulated with 818‐nm gold nanorods and the size of these AuNDs was mainly ranged from around 200 to 400 nm in diameter (Figure 4a,b). The average concentration of AuNDs after production was 4.16±0.73×10^11^/ml (mean±SD). To improve the interaction between AuNDs and BNL cells, we conjugated the AuNDs with FITC‐CD54, which is a green‐fluorescence‐conjugated surface protein marker of BNL cells, to facilitate the attachment of AuNDs to the cells. After the conjugation, we found that 67% of AuNDs were successfully conjugated with FITC‐CD54 detected by the flow cytometry (Figure 4c). To further confirm the ability of FITC‐CD54‐conjugated AuNDs to recognize and attach to the BNL cells, we took the fluorescent images after incubation of cells and conjugated AuNDs, and it was found that the green fluorescence was located on the surface of most of the cells indicating that conjugated AuNDs were successfully attached on the cell membrane (Figure 4d).

### 3.2 Vaporization and cavitation effects affected by laser activation number

**FIGURE 5:**
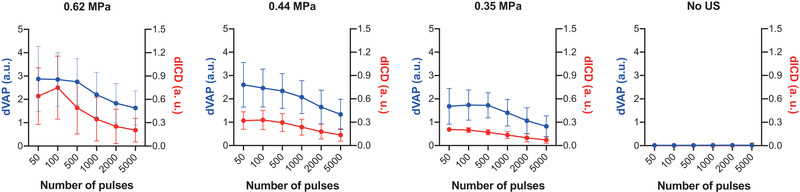
Differential vaporization dose (dVAP) and differential inertial cavitation dose (dICD) values as a function of different activation firing numbers. Blue and red lines indicate the dVAP and dICD values, respectively. The laser pulse repetition frequency (PRF) was set as 20 Hz and the US cycle was set as 10 cycles in all experiments. Symbols and error bars denote the mean and SD from six individual experiments

To achieve the goal of inducing repeated vaporization with the same batch of AuNDs, a lowering inertial cavitation effect is required so that the destruction of AuNDs can be minimized within the activation period. We firstly examined how accumulated firings (represented by the activation number, i.e., the number of laser pulses) determine the vaporization effect of AuNDs. We applied 5000 firings with the laser fluence and the pulse repetition frequency (PRF) that were used in our previous study (i.e., 12.02 mJ/cm^2^, PRF = 20 Hz).^15^ Both dVAP and dICD were analyzed among various activation firing sets (Figure 5). First, we applied different activation firings from 50 to 5000 with three different PNPs or without US stimulation. It was found that both dVAP and dICD values were reduced with the decrease of PNP in the groups with US stimulation, and were almost not detectable in the group without US stimulation. The correlation between dVAP and dICD was high in the three groups with US stimulation, where the Pearson's coefficient is 0.94 (*p* <0.01), 0.99 (*p* <0.001), and 0.96 (*p* <0.01) for the group with a PNP of 0.62, 0.44, and 0.35 MPa, respectively. Generally, in all of the three groups with US stimulation, both dVAP and dICD values decreased with increasing activation firings. In all cases with US stimulation, as the number of activation firings increases, both dVAP and dICD decrease. Nonetheless, depending on the specific PNP values, the dVAP can maintain at a relatively stable level for a period of time (e.g., for PNP of 0.35 MPa and within the first 500 activation firings). This indicates that it is possible to use a relatively low PNP to avoid rapid microbubble destruction while producing repeated vaporization effects for sonoporation.

**FIGURE 6:**
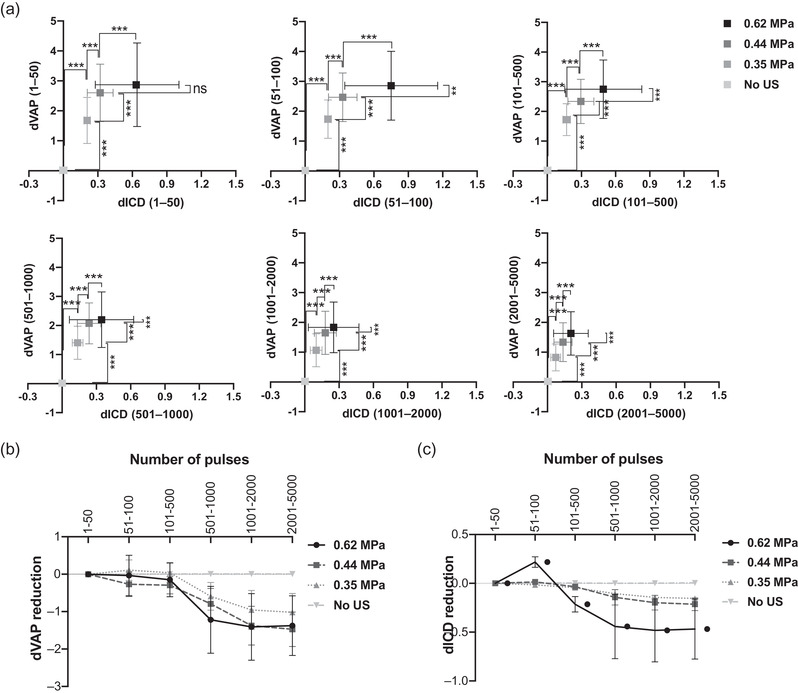
Vaporization and cavitation effect in different activation firing sections. (a) differential vaporization dose (dVAP) as a function of differential inertial cavitation dose (dICD) in different activation sections. (b, c) Reduction of dVAP and dICD as a function of different activation firing sections within 5000 firings. Symbols and error bars denote the mean and standard deviation (SD) from six individual experiments. The student's *t*‐test was applied for the determination of the significant difference between two data sets. *, *p* < 0.05; **, *p* < 0.01; ***, *p* < 0.001

We then explored how vaporization and inertial cavitation events occurred over the period of a total of 5000 activation firings. The dVAP and dICD were measured with different levels of PNPs under the laser fluence of 12.02 mJ/cm^2^. In Figure 6, the activation firings were broken down into the following sections: 1–50, 51–100, 101–500, 501–1000, 1001–2000, and 2001–5000. In Figure 6a, dVAP and dICD are plotted with the *p*‐values between two adjacent PNPs from Student's *t*‐test. For repeated vaporization, generally, the dVAP is desired to increase while the dICD is desired to remain at a similar level when increasing the PNP. As shown in Figure 6, the 0.44 MPa group has a consistently high level of dVAP (i.e., significant vaporization) while maintaining a relatively low level of dICD (i.e., less microbubble destruction). Therefore, we found that the PNP of 0.44 MPa is desirable among all PNPs. The reduction of dVAP and dICD values were also analyzed, and all values were subtracted from the values calculated from the first 50‐firings section. As shown in Figures 6b,c, both dVAP and dICD decreased more obviously after certain number of activation firings. In addition, very few vaporization or inertial cavitation events were found if US was not applied (i.e., “No US” group). Moreover, the reduction of dVAP values collected from all groups with US stimulation show that the reduction reached a plateau after the laser was activated over 2000 times, and the reduction of dICD values appeared to reach plateaus after the activation was over 1000 times in 0.62 MPa group and 2000 times in 0.44 and 0.35 MPa groups. It indicates that marginal vaporization and inertia cavitation effects were minimal after certain numbers of activation firings. Therefore, under the same driving optical energy, when the given US PNP was lower than 0.62 MPa and the activation numbers was less than 2000 times may be preferred for major vaporization effects. In other words, it indicates that if we want to attain repeated vaporization, we should set the US PNP smaller than 0.62 MPa.

### 3.3 Sonoporation and activation firings

**FIGURE 7:**
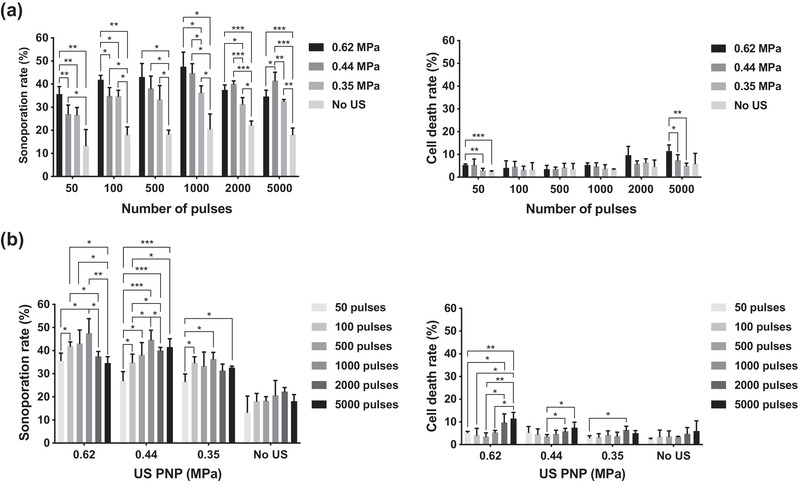
Sonoporation as a function of activation firing and ultrasound peak negative pressure (US PNP). (a) Sonoporation rate and cell death rate grouped with different activation firings. (b) Sonoporation rate and cell death rate grouped with different US PNPs. The laser pulse repetition frequency (PRF) was set as 20 Hz and the number of US cycles was set at 10 cycles in all experiments. Each column indicates the mean and standard deviation (SD) from six individual experiments. The student's *t*‐test was applied for the determination of the significant difference between two data sets. *, *p* < 0.05; **, *p* < 0.01; ***, *p* < 0.001

We next explored how activation firing numbers affect cellular sonoporation rate under different US PNP levels and activation numbers with a fixed laser fluence of 12.02 mJ/cm^2^. We first compared the sonoporation events occurring under different levels of PNPs with a certain given activation firing number (Figure 7a). When comparing the samples treated with the US to samples without US treatment, a significant enhancement of sonoporation rate was found in almost all samples treated with the US except the samples treated with PNP of 0.35 MPa and 50 activation firings. In the groups with low activation firings (i.e., 50 and 100 firings), the sonoporation rate was significantly increased when PNP increased from 0.44 to 0.62 MPa. While in the groups with 1000 or more activation firings, the sonoporation rate in most of the groups had no significant enhancement between 0.44 and 0.62 MPa. When PNP increased from 0.35 to 0.44 MPa, only delivering 1000 or more activation firings to samples could significantly enhance the sonoporation rate. It was noteworthy that in the group with 5000 activation firings, the sonoporation rate became significantly reduced when the PNP increased from 0.44 to 0.62 MPa, while the associated cell death rate was significantly increased.

To further clarify if the sonoporation rate was affected by different activation firing times, we grouped the data with different US PNPs (Figure 7b). It was found that without US stimulation, the sonoporation rate was only around 20% in all groups, and there was no significant change regardless of the activation numbers. In the groups of 0.44 and 0.62 MPa, the sonoporation rate gradually increased with the increased activation numbers from 50 to 1000 firings and was significantly reduced when the firing numbers were above 1000. And the groups with the reduced sonoporation rate (i.e. 2000‐ and 5000‐firings groups) had significantly high cell death rates. For the group of 0.35 MPa, there was no significant change of sonoporation rate when the firing numbers were above 100, and the cell death rate stayed around the baseline level (i.e., smaller than 5%).

Comparing the groups with the lowest sonoporation rate to the group with the highest one (i.e., the groups with the activation number of 50 vs. 1000), the increment of sonoporation rate was 11.9% for the 0.62 MPa group, 17.7% for the 0.44 MPa group, 9.7% for 0.35 MPa group, and 7.3% for the group without US stimulation. It indicates that the 0.44 MPa group displayed a better enhancement of the sonoporation effect. The sonoporation rate obtained from 0.62 and 0.44 MPa groups was comparable, it further indicates that no more enhancement could be achieved when US PNP was larger than 0.44 MPa and the given activation number was set as 1000. Thus, we fixed the given activation number at 1000 for further examination on the sonoporation effect. In summary, we demonstrated that the sonoporation rate can be improved while maintaining the cell death rate at the baseline level when the laser fluence and the US PNP was set as 12.02 mJ/cm^2^ and 0.44 MPa, respectively, in combination with the activation firing being set at or below 1000 times. Such conditions are consistent with the preferred parameters for repeated vaporization that were previously discussed.

### 3.4 Sonoporation effect affected by laser fluence and acoustic pressure

**FIGURE 8:**
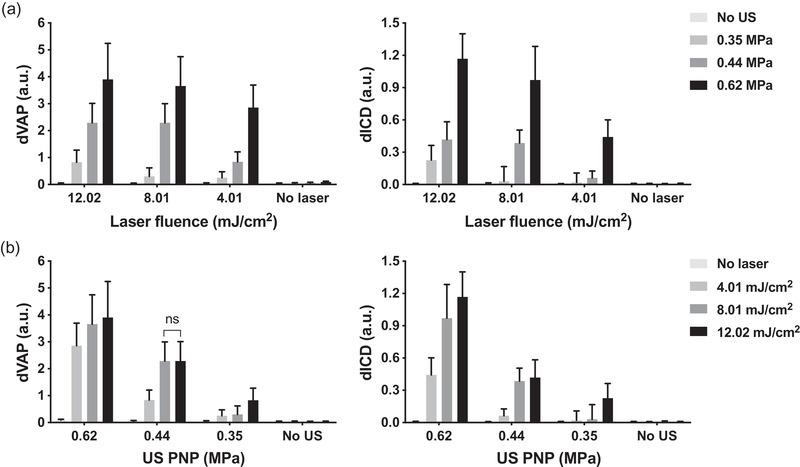
differential vaporization dose (dVAP) and differential inertial cavitation dose (dICD) as a function of laser fluence and ultrasound peak negative pressure (US PNP). The activation number was fixed at 1000, laser pulse repetition frequency (PRF) was set as 20 Hz, and the number of US cycles was set at 10 in all experiments. Each column indicates the mean and SD from six individual experiments. The student's t‐test was applied for the determination of the significant difference between two data sets. ns, no significance. Except for the data set labeled with no significant difference and data sets with no US or laser treatment, all other data sets showed a significant difference

To explore the preferred optical and acoustic parameters to induce sonoporation when the activation number was fixed at 1000 firings, we investigate the sonoporation effect in terms of different laser fluences and acoustic pressures. We first analyzed the dVAP and dICD values measured under different laser fluences and acoustic pressures. The results showed that the level of both dVAP and dICD followed the strength of the given laser fluences and acoustic pressures (Figure 8). In other words, both dVAP and dICD values were significantly enhanced when increasing the laser fluence or the acoustic pressure. It indicates that when the activation number was fixed at 1000 firings, the vaporization effect and inertial cavitation effect are highly correlated with the given laser fluences and acoustic pressures.

**FIGURE 9:**
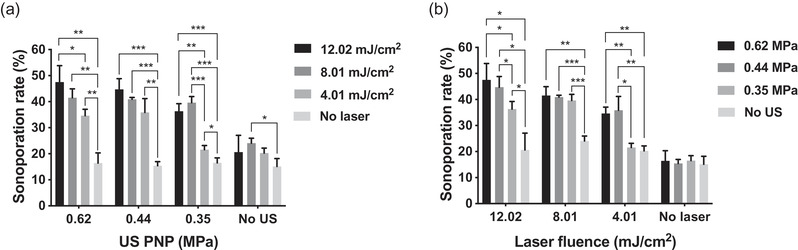
Sonoporation rate as a function of (a) ultrasound peak negative pressure (US PNP) or (b) laser fluence. The activation number was fixed at 1000, laser pulse repetition frequency (PRF) was set as 20 Hz, and the number of US cycles was set at 10 cycles in all experiments. Each column indicates the mean and standard deviation (SD) from six individual experiments. The student's *t*‐test was applied for the determination of the significant difference between two data sets. *, *p* < 0.05; **, *p* < 0.01; ***, *p* < 0.001

After measuring the sonoporation rate induced under these parameters, it was found that both ADV and ODV are needed for effective sonoporation. ADV or ODV alone does not induce significant sonoporation (Figure 9). When we grouped the data by different levels of US PNP, once the given laser fluence was above 4.01 mJ/cm^2^, the sonoporation rate can be significantly induced even the given US PNP was only 0.35 MPa, but a more significant enhancement of sonoporation rate was induced if the laser fluence was increased to 8.01 mJ/cm^2^, and the sonoporation rate of 8.01 and 12.02 mJ/cm^2^ group was comparable (Figure 9a). When the given US PNP was above 0.44 MPa, the sonoporation rate was almost not enhanced at all laser fluences (Figure 9a). Significant enhancement of the sonoporation effect was found when the given US PNP was increased from 0.35 to 0.44 or 0.62 MPa in the 4.01 and the 12.02 mJ/cm^2^ groups (Figure 9b). But in the 8.01 mJ/cm^2^ group, the sonoporation rate of the samples measured from three different levels of US PNP was comparable to each other (Figure 9b). Based on these results, the minimal acoustic and optical energy for inducing a high level of sonoporation rate (around 40 % in our case) can be determined as 0.35 MPa of US PNP combined with 8.01 mJ/cm^2^ of laser fluence or 0.44 MPa of US PNP combined with 4.01 mJ/cm^2^ of laser fluence.

### 3.5 Vaporization‐based sonoporation

**FIGURE 10:**
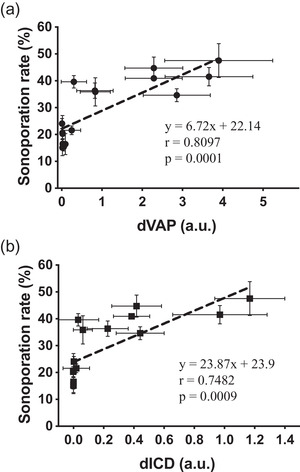
Correlation of sonoporation rate and differential vaporization dose (dVAP)/ differential inertial cavitation dose (dICD) values. The correlation coefficient (*r*), the *p*‐value of the Pearson's correlation test, and the equation of the linear regression were shown below the lines. Symbols and error bars denote the mean and standard deviation (SD) from six individual experiments

To further examine the possibility for vaporization‐based sonoporation, we analyzed the correlation between sonoporation rate and dICD or dVAP values collected when the activation number was fixed at 1000 (Figure 10). The analysis showed that both dVAP and dICD values were positively correlated with induced sonoporation rate. The higher correlation between sonoporation rate and dVAP suggested that the vaporization in our experiments had a higher possibility to induce sonoporation.

**FIGURE 11:**
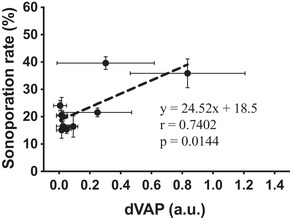
Correlation of sonoporation rate and differential vaporization dose (dVAP) values from conditions without significant inertial cavitation. The correlation coefficient (*r*), the *p*‐value of the Pearson's correlation test, and the equation of the linear regression were shown below the line. Symbols and error bars denote the mean and standard deviation (SD) from six individual experiments

According to the results shown in Figures 8 and 9, we defined the sonoporation rate detected when cells were not treated with US and laser as the background sonoporation values, we found the minimal dICD value for significant induction of sonoporation was 0.13. In order to analyze whether the positive correlation still exists without the inertial cavitation effect, we thus selected the samples with dICD values lower than 0.1 to represent the samples without effective inertial cavitation effect for inducing sonoporation. According to the analysis, a high positive correlation still exists (Figure 11). The result further displayed the possibility that without the inertial cavitation effect, vaporization of AuNDs can also effectively induce sonoporation.

## 4 DISCUSSION

The energy for inducing inertial cavitation was found larger than that required for vaporization. Violent collapse of microbubbles (i.e., inertial cavitation) also has proved to be a factor for evoking cell death. With the aim of reducing driving energy and the consumption of AuNDs, in this study, we made an effort to leverage the repeatability of the vaporization/recondensation cycle to achieve effective sonoporation. Increasing activation firings can enhance both inertial cavitation and sonoporation.^35^, ^36^, ^37^ However, we found that as more activation numbers were given, lower vaporization and inertial cavitation effects were observed (Figure 5). This suggests that additional activation firings result in the destruction of AuNDs. Within 5000 activation firings, both the vaporization and the inertial cavitation gradually reduced over the whole activation process, and the reduction held after the activation was over 2000 times (Figure 6). But in the group with a higher US PNP (i.e., 0.62 MPa), the reduction of inertial cavitation effect appeared to reach a plateau after the activation was over 1000 times, suggesting a higher US PNP tends to induce the inertial cavitation effect (the destruction of vaporized AuNDs) earlier even as the vaporization of AuNDs was still growing, and these newly grown vaporized AuNDs may be generated from AuNDs with smaller size so that their inertial cavitation threshold may be higher than 0.62 MPa.

As shown in Figure 7, both the acoustic pressure and the activation number have a role in regulating the sonoporation effect. The highest mechanical index (MI) displayed in our study was 0.62, which is lower than that in other reports regarding the study of ADV of PFP droplets.^5^, ^9^, ^10^, ^35^ Among these cases, the smallest MI applied for inducing ADV is 0.7, and their size of the droplets is 0.89 μm,^10^ and the MI for inducing ADV of the largest size of droplets (i.e., 1.4–2 μm) is 0.81 and 0.95 by using the transmit frequency of 0.74 and 1.1 MHz, respectively, combined with a relatively long pulse duration (e.g., 100 ms).^35^ The AuNDs used in the study were much smaller than the droplets used in the two studies in the literature, where the average size was only around 0.35 μm, thus it is reasonable that no obvious vaporization events were observed when we did not apply the US on AuNDs. Even we increased the acoustic pressure to 0.62 MPa (i.e., MI is equal to 0.62), it still required optical stimulation to trigger vaporization. Although cells responding to larger acoustic pressures can generally increase the sonoporation rate, when higher activation numbers were delivered, the cell death rate significantly increased. The possible reason for increased cell death may be due to failed resealing of pores on the cell membrane. It was found that after cells were sonoporated, the generated pores on the cell membrane would be resealed after a few to few tens of seconds for survival.^36^, ^38^ In our case, the PRF was 20 Hz so the time for completing 5000 activation firings was 250 s, thus failing to reseal might be possible because the vaporization and inertial cavitation kept generating pores or enlarging pores during this period of time. In addition, it has been demonstrated that increasing activation firings and duty cycles induce cell death during the sonoporation process.^39^, ^40^, ^41^ Continuous inertial cavitations have also been reported to induce the production of reactive oxygen species or suddenly evoke calcium influx to cause cell death.^29^, ^32^ Accordingly, the optical and acoustic parameters need to be carefully selected to achieve effective sonoporation while minimizing cell death.

The level of laser fluence was also a critical factor to induce sonoporation,^15^ we found that higher laser fluence induced a higher sonoporation rate but no significant enhancement was observed when laser fluence was above 8.01 mJ/cm^2^ (Figure 9). Combining with the analysis of sonoporation rate as a function of US PNPs, it indicates that the sonoporation effect was saturated when the laser fluence reached 8.01 mJ/cm^2^ and US PNP reached 0.44 MPa. In comparison with our previous study,^15^ through adjusting the activation number to 1000 firings, a similar sonoporation rate (around 40 %) can be successfully achieved by using smaller acoustic and optical energy, where 0.35 MPa of US PNP combined with 8.01 mJ/cm^2^ of laser fluence and 0.44 MPa of US PNP combined with 4.01 mJ/cm^2^ of laser fluence can achieve 39.64±2.32% and 35.88±5.28% of sonoporation rate, respectively. Although we have found the energy threshold of inducing sonoporation, the determining factors for triggering sonoporation still remain unclear. Some models have been proposed to explain this effect according to the investigation from high‐speed imaging,^25^, ^42^ it is still hard to clearly capture fast NDs dynamics without a sufficiently high sampling rate during imaging.

The ambient temperature issue should be also considered. When the ambient temperature is 37°C, the threshold of laser fluence for inducing ODV of PFP droplets can be reduced to 4–5 mJ/cm^2^,^43^, ^44^ but it can increase to 100 mJ/cm^2^ when the ambient temperature is only 25°C.^45^ According to previous reports in the literature, repeated vaporization is easier to achieve when the ambient temperature is lower than the droplet core PFC boiling point.^8^, ^20^ In our experiments, the ambient temperature in the media is 25°C and the boiling point of PFP is 29°C so repeated vaporization is achievable. However, concerning the human body temperature is 37°C, using PFC with a higher boiling point as the ND core and conducting the experiments at 37°C to improve vaporization‐based sonoporation should be considered in future studies. The threshold of laser fluence for droplets vaporization can also be further reduced through mixing with different core materials or modifying the optical absorbing material structure.^12^, ^43^, ^46^, ^47^, ^48^

## 5 CONCLUSIONS

Simultaneous optical and acoustic stimulation has proven to be able to synergistically enhance the sonoporation effects. In this study, by analyzing vaporization and inertial cavitation from different laser/US firing combinations, we found that the activation numbers and the selective section of laser/US activation play key roles in determining the vaporization, inertial cavitation, and sonoporation effects. We tested the hypothesis and demonstrated that sonoporation can be effectively induced mainly by repeated vaporization instead of inertial cavitation. With this approach, both the required acoustic and optical energy were smaller than those applied in previous studies. With lower applied energy and less inertial cavitation, vaporization‐based sonoporation can improve drug delivery efficiency and safety during therapy. Decreased consumption of NDs can also reduce the air embolism caused by overloaded contrast agents, and further enhance theragnosis by leveraging the improved imaging performance.

## CONFLICT OF INTEREST

The authors declare no conflict of interest.

## Data Availability

The data that support the findings of this study are available from the corresponding author upon reasonable request.
